# Action of vanillin (*Vanilla planifolia*) on the morphology of tibialis anterior and soleus muscles after nerve injury

**DOI:** 10.1590/S1679-45082017AO3967

**Published:** 2017

**Authors:** Ana Luiza Peretti, Juliana Sobral Antunes, Keli Lovison, Regina Inês Kunz, Lidyane Regina Gomes Castor, Rose Meire Costa Brancalhão, Gladson Ricardo Flor Bertolini, Lucinéia de Fátima Chasko Ribeiro

**Affiliations:** 1 Universidade Estadual do Oeste do Paraná, Cascavel, PR, Brazil.

**Keywords:** Muscle, skeletal, Muscle denervation, Peripheral nerve injuries, Rehabilitation, Biological products

## Abstract

**Objective:**

To evaluate the action of vanillin (*Vanilla planifolia*) on the morphology of tibialis anterior and soleus muscles after peripheral nerve injury.

**Methods:**

Wistar rats were divided into four groups, with seven animals each: Control Group, Vanillin Group, Injury Group, and Injury + Vanillin Group. The Injury Group and the Injury + Vanillin Group animals were submitted to nerve injury by compression of the sciatic nerve; the Vanillin Group and Injury + Vanillin Group, were treated daily with oral doses of vanillin (150mg/kg) from the 3rd to the 21st day after induction of nerve injury. At the end of the experiment, the tibialis anterior and soleus muscles were dissected and processed for light microscopy and submitted to morphological analysis.

**Results:**

The nerve compression promoted morphological changes, typical of denervation, and the treatment with vanillin was responsible for different responses in the studied muscles. For the tibialis anterior, there was an increase in the number of satellite cells, central nuclei and fiber atrophy, as well as fascicular disorganization. In the soleus, only increased vascularization was observed, with no exacerbation of the morphological alterations in the fibers.

**Conclusion:**

The treatment with vanillin promoted increase in intramuscular vascularization for the muscles studied, with pro-inflammatory potential for tibialis anterior, but not for soleus muscle.

## RESUMO

**Objetivo:**

Avaliar a ação da vanilina (*Vanilla planifolia*) sobre a morfologia dos músculos tibial anterior e sóleo após lesão nervosa periférica.

**Métodos:**

Ratos Wistar foram divididos em quatro grupos, com sete animais cada, sendo Grupo Controle, Grupo Vanilina, Grupo Lesão e Grupo Lesão + Vanilina. Os animais dos Grupos Lesão e Grupo Lesão + Vanilina foram submetidos à lesão nervosa por meio da compressão do nervo isquiático, e os Grupos Vanilina e Grupo Lesão + Vanilina foram tratados diariamente com doses orais de vanilina (150mg/kg) do 3^o^ ao 21^o^ dia após a indução da lesão nervosa. Ao término do experimento, os músculos tibial anterior e sóleo foram dissecados e seguiram o processamento de rotina em microscopia de luz, para posterior análise morfológica.

**Resultados:**

A compressão nervosa promoveu alterações morfológicas características de denervação, sendo que o tratamento com vanilina foi responsável por respostas distintas nos músculos estudados. Para o tibial anterior, houve aumento do número de células satélites, núcleos centrais e atrofia das fibras, bem como desorganização fascicular. Já no sóleo, houve apenas aumento da vascularização, sem exacerbação das alterações morfológicas nas fibras.

**Conclusão:**

O tratamento com vanilina promoveu o aumento da vascularização intramuscular para os músculos estudados, com potencial pró-inflamatório para o tibial anterior, o que não ocorreu no músculo sóleo.

## INTRODUCTION

Peripheral nerve injuries (PNI) occur worldwide and can generate physical and functional changes, commonly associated with pain and sequelae, and also socioeconomic impacts.^1,2^ The main etiological factors of this type of lesion are road accidents, knife or gunshot wounds, falls, and compression or crushing of nervous tissue. The sciatic nerve is the most frequently affected nerve in peripheral nerve injuries of lower limbs.^1,3^

In complete lesions, the interruption of neuromuscular transmission causes immediate loss of muscle voluntary/reflex activity, alters excitability of the membrane, decreases its resting potential, and renders the muscle fibers hypersensitive to acetylcholine.^4^ Some morphological changes, such as atrophy, degeneration and necrosis, as well as decreased strength and replacement by fibrous connective tissue, may also occur.^5,6^

Among the treatments available for nerve and musculoskeletal dysfunctions, we highlight those performed in a multidisciplinary way, considering the elimination of risk factors and including physical therapy, drug therapy and surgery, which is indicated only in cases of severe neurological impairment, significant pain, or complete failure of medical treatment.^7^ As disabling pain is a frequent outcome, PNI treatments are almost always associated with the use of some type of medication, mainly analgesics and anti-inflammatory agents.^8^

The use of medications is often effective in relieving pain quickly, but there is always a risk of adverse reactions. Hence the search for new drugs is indispensable, and among them, natural products with a potential analgesic effect and fewer adverse side effects have been investigated.^8^ Based on this premise, vanillin, a chemical compound extracted from the pods of (*Vanilla planifolia*), a tropical orchid, has been experimentally studied in the treatment of neuropathic pain, allodynia and edema, with positive effects in the treatment of nerve compression symptoms.^9,10^

Despite its use, there is no consensus in the literature about the effects of vanillin on PNI. In addition, since neural and musculoskeletal systems are closely related, the therapeutic management should include not only the nerve but also the innervated muscle.

## OBJECTIVE

To evaluate the effects of vanillin (*Vanilla planifolia*) on the morphology of tibialis anterior and soleus muscles after peripheral nerve injury.

## METHODS

This study was conducted at the Laboratory of Physical Injury and Physiotherapeutic Resources of the *Universidade Estadual do Oeste do Paraná*. All procedures adopted were previously approved by the Animal Ethics Committee of the university, according to international standards for animal experimentation.

### Sample groups

The sample size calculation for this study was based on the researchers’ experience in studies of this nature, as well as on data described in the literature, regarding the experimental effects of vanillin.^9,10^

A total of 28 male Wistar rats were used, with mean age of 10 weeks and weight of 350g, kept in standard polypropylene boxes, at room temperature of 23±1°C and a 12-hour photoperiod, receiving water and food *ad libitum*.

The animals were randomly divided into four groups of seven rats each, as follows: Control Group (CG), composed of animals that did not undergo any intervention; Vanillin Group (VG), composed of animals without nerve compression, but treated with vanillin; Injury Group (IG), with animals submitted to sciatic nerve compression, but without therapy; and Injury + Vanillin Group (IVG), with animals submitted to sciatic nerve compression and treated with vanillin.

### Injury protocol

Before surgical sciatic nerve compression, the animals were weighed and intraperitoneally anesthetized with ketamine hydrochloride (50mL/kg) and xylazine hydrochloride (10mL/kg). After shaving the right pelvic limb and performing local asepsis with 70% alcohol, an incision was made parallel to the fibers of the biceps femoralis muscle, exposing the sciatic nerve, which was compressed with a hemostatic forceps for 30 seconds. The clamping pressure was standardized for all animals and always performed by the same researcher, grasping the second notch of the forceps as a reference.^3^ Finally, suture by planes and iodine application were performed on the incision.

### Vanillin administration protocol

The administration of vanillin to VG and IVG was started on the third postoperative day (PO). The animals of these groups received daily oral doses of 150mg/kg of vanillin, by gavage, until the PO 21th.

### Morphologic analysis

On the 22nd day of the experiment, the animals of all groups were weighed, anesthetized, and euthanized by guillotine decapitation. The right tibialis anterior and soleus muscles were dissected, fixed in Metacarn solution for 24 hours, and stored in 70% alcohol. Subsequently, the muscles underwent routine histological processing, for paraffin embedding, and were stained with hematoxylin and eosin and Mallory trichrome.

The slides obtained were analyzed under a light microscope and photomicrography. Ten images of each muscle were randomly obtained, and one hundred muscle fibers were measured for the smallest diameter and area of the fiber, using the Image Pro-Plus 6.0 software. The analysis of the connective tissue, endomysium and perimysium was performed by percentage of pixels, using the GIMP 2.0 software.

### Analysis of results

The normality of the data was verified by the Shapiro-Wilk test, which was later compared with the unidirectional Analysis of Variance (ANOVA) test, and Tukey post-test using the Graph Pad Prism 6.0 software. For all analyzes, p<0.05 was considered statistically significant.

## RESULTS

**Figure 1:**
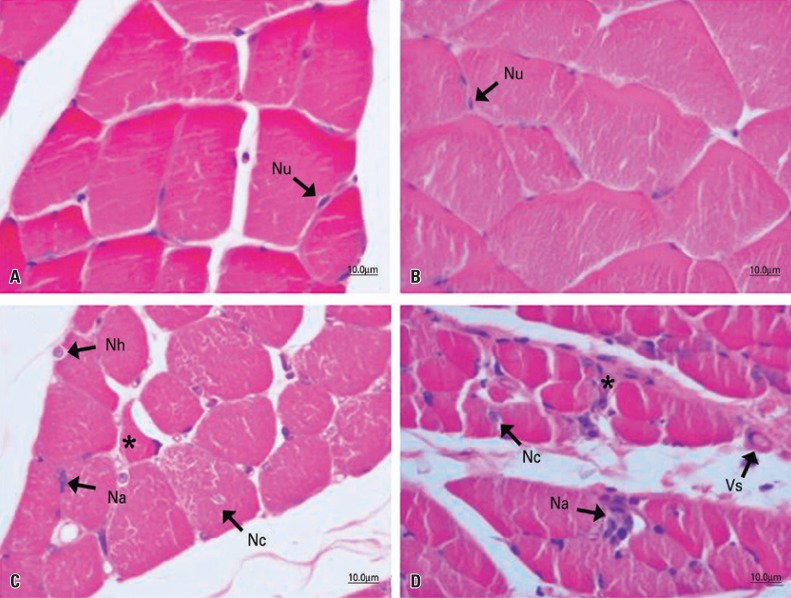
Photomicrographs of the tibialis anterior muscle of Wistar rats, transverse section, hematoxylin and eosin staining. Control Group (A), Vanillin Group (B), Injury Group (C), and Injury + Vanillin Group (D). In A and B, fascicular organization with polygonal fibers and peripheral nuclei (Nu). In C, polymorphic muscle fibers (*); hypertrophic (Nh), central (Nc), or agglomerated (Na) nuclei at the periphery of the fiber. In D, polymorphic fiber (*); central (Nc) and agglomerated (Na) nuclei at the periphery of the fiber; and dilated blood vessels (Vs)

**Figure 2:**
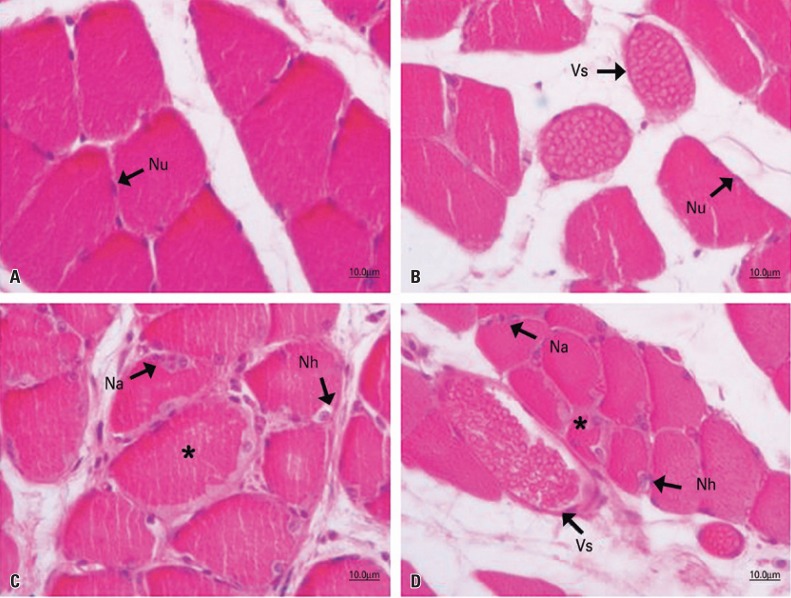
Photomicrographs of the soleus muscle of Wistar rats, transverse section, hematoxylin and eosin staining. Control Group (A), Vanillin Group (B), Injury Group (C), and Injury + Vanillin Group (D). In A, fascicular organization with polygonal fibers and peripheral nuclei (Nu). In B, tissue organization similar to the Control Group, with dilated blood vessels (Vs). In C, muscle fibers with color change (*), hypertrophic nuclei (Nh) and agglomerated nuclei (Na) at the periphery of the fiber. In D, regenerating polymorphic fiber (*); hypertrophic (Nh) and agglomerated (Na) nuclei at the periphery of the fiber; and dilated blood vessels (Vs)

In the analysis of the tibialis anterior muscle (Figure 1A) and the soleus muscle (Figure 2A) of the CG animals, a normal aspect was observed, with the fibers organized in a fascicular pattern and individually in a polygonal and multinucleate form, whose nuclei were located peripherally, near the sarcoplasmic membrane. The same morphology was observed in the tibialis anterior muscle (Figure 1B) of the VG animals, which were not injured and were treated with vanillin. In the soleus muscle of the animals of this group, the aspect of muscle fibers was normal, but an increased vascularization was observed, marked by both dilated and turgid blood vessels (Figure 2B).

In IG, muscle fibers from both muscles - tibialis anterior (Figure 1C) and soleus - (Figures 2C) had typical changes resulting from nerve compression. In these animals, there were a marked fascicular disorganization and alteration in the size of fibers indicating atrophy, in addition to some fibers with loss of the characteristic polygonal shape. Muscle fibers in advanced degenerative process were sparsely observed, although most of them had hypertrophic nuclei located in the center of the fiber or peripherally, with or without a basophilic halo, as well as agglomerated and clustered nuclei in the periphery of the fiber – a region that occasionally presents color changes.

In the animals submitted to nerve compression and treated with vanillin IVG, differences in the response of the two muscles were observed. In the tibialis anterior muscle, the fibers had more pronounced morphological injury characteristics when compared to IG, with an increase in the number of satellite cells, central nuclei and a decrease in fiber size, as well as greater disorganization and fascicular degeneration. In these animals, some injured muscle fibers were observed, some of them in degeneration and others already in regeneration, associated to a large number of blood capillaries, which may indicate neovascularization (Figure 1D). In the soleus muscle, the same characteristics of IG were observed, and there was also an extensive increase in vascularization, with dilated and turgid blood vessels (Figure 2D).

Nerve compression reduced the fiber cross-sectional area of the two muscles studied. In the tibialis anterior muscle, there was a decrease in area in IG and IVG when compared to CG (p<0.01) and VG (p<0.001). In the soleus muscle, the fiber cross-sectional area was smaller in IG and IVG when compared to CG (p<0.05; p<0.01) and VG (p<0.05; p<0.01), and the treatment with vanillin did not affect this parameter (Table 1).

**Table 1:** Morphometric parameters of the tibialis anterior and soleus muscles

Parameters	Muscles	Groups			

		CG	VG	IG	IVG
Cross-sectional area (µm)	Tibialis anterior	2042.2±438.6	2232.3±452.4	1430.8±286.7*^†^	1310.7±208.6*^†^
	Soleus	2550.4±793.8	2584.4±188.3	1789.2±311.9*^†^	1524.7±307.8*^†^
Smaller diameter (µm)	Tibialis anterior	41.2±4.95	41.4±4.12	33.8±2.67*^†^	33.1±2.28*^†^
	Soleus	44.3±8.40	44.1±2.89	39.4±3.50	34.6±4.25*^†^
Connective tissue (%)	Tibialis anterior	2.8±0.25	3.0±0.82	3.4±0.53	5.2±1.67*^†‡^
	Soleus	7.9±0.70	6.6±2.03	6.8±2.09	7.0±1.36*^†‡^

Unidirectional Analysis of Variance (ANOVA) test, and Tukey post-test. * different from CG; ^†^ different from VG; ^‡^ different from IG.

CG: Control Group; VG: Vanillin Group; IG: Injury Group; IVG: Injury + Vanillin Group.

As for the lower diameter of the muscle fiber, nerve injury also caused a decrease in the parameter analyzed in the tibialis anterior muscle, with differences in IG and IVG as compared to CG (p<0.01; p<0.001) and VG (p<0.01; p<0.001). In the soleus muscle, there was a reduction in the fiber diameter of IVG animals when compared to CG (p<0.01) and VG (p<0.01).

In the anterior tibial muscle, there was an increase in the amount of intramuscular connective tissue of IVG animals when compared to the other groups (p<0.001). In the soleus muscle, there was no significant difference in the amount of connective tissue among the groups.

## DISCUSSION

The sciatic nerve compression model used in this study can be described as an axonotmosis type of injury,^11,12^ which leads to an interruption of neuromuscular transmission. Morphometric changes were observed in the tibialis anterior and soleus muscles of IG animals, with a reduction in the cross-sectional area and a smaller diameter of the muscle fiber. This set of characteristics is common in nerve injuries and may be accompanied by functional disabilities.^13-15^

The morphological alterations observed in IG animals confirm that nerve injury caused interruption of neuromuscular transmission. Muscle fibers with irregular contours and centralized nuclei were also observed by Polônio et al.,^16^ in the tibialis anterior muscle of rats submitted to a complete section of the sciatic nerve; they characterize a set of biochemical and mechanical changes in the fibers, which may result in various degrees of functional loss.

The oral administration of vanillin in the VG animals did not alter the morphometric and morphological parameters of the fibers of the studied muscles, although there was an increase in the vascularization of the soleus muscle, evidenced by greater quantity of blood vessels, frequently dilated. In this sense, the present study contrasts with the results of some authors, who pointed out the antiangiogenic potential of vanillin,^17-19^ but it is corroborated by Raffai et al.,^20^ who proposed that vanillin promotes arterial relaxation by inhibiting Ca^2^ channels, preventing the influx of Ca^2^+ into the cell, that is, it has vasodilating properties.

In addition to its antiangiogenic and vasodilating potential, other properties have already been described for vanillin, such as antioxidant, anti-inflammatory, antiproliferative, and also antitumor actions.^18,19,21^ Its biocompatibility associated with its anti-inflammatory effect could make vanillin a promising compound. In the present study, there were distinct responses in the tibialis anterior and soleus muscles to the oral ingestion of vanillin in animals submitted to a previous lesion of the sciatic nerve.

Three weeks after nerve injury, the muscle fibers are still involved in the regeneration process,^22^ a stage in which inflammatory mediators are present in the tissue. In the tibialis anterior muscle, the treatment with vanillin caused an intensification of the inflammatory response, as there was a worsening of the tissue morphology when compared to IG animals. In the soleus muscle, it can be inferred that, despite the greater number of blood vessels, vanillin did not have a positive or negative effect on the morphology of the fibers, which resembled those of IG.

In the initial phase of muscle injury, neutrophils rapidly invade the lesion site and promote the repairing process by delivery of cytokines, which attract and activate additional inflammatory cells.^22,23^ According to Tidball,^24^ neutrophils may exacerbate prior injuries due to the release or generation of reactive oxygen species (ROS), which can lead to loss of cellular integrity and even fiber necrosis.^25^ Under normal conditions, ROS are eliminated by antioxidant systems present in muscles. According to Muller et al.,^26^ muscles composed of greater amount of type II fibers, such as the tibialis anterior, are more susceptible to oxidative damages than type I fibers, which have a slow contraction, such as the soleus muscle. Therefore, vasodilation and apparent neovascularization caused by the treatment with vanillin may have caused greater release of inflammatory mediators, such as neutrophils, which caused oxidative stress in the tibialis anterior muscle, exacerbating the injury process.

Furthermore, according to Csete et al.,^27^ the behavior of satellite cells can be modified by concentration of oxygen in the tissue, through processes mediated by the generation of ROS.^28^ Recently, in an *in vitro* study, researchers demonstrated that myoblast proliferation and differentiation are negatively influenced by increased oxygen concentration.^29^ Therefore, the increased blood flow generated by vanillin caused greater oxygen concentration in tissues, which may have hindered the normal process of muscle regeneration through the activation of satellite cells, so that the tibialis anterior muscle still presented large amounts of fibers in a degenerative process.

In the present study, it was found that the treatment with vanillin promotes its effects by altering tissue blood flow, markedly by intramuscular vasodilation. As a limitation, we believe that specific methodologies for vascular assessment are important to elucidate definitively the effects of vanillin. Studies should be conducted in different muscle groups to clarify whether its effects are actually related to the type of fiber that composes the greatest proportion of the muscle. It should be noted that studies on the effects of vanillin *in vivo* are scarce in the literature, and further studies are required before indicating the substance as a treatment.

## CONCLUSION

Compression of the sciatic nerve causes morphological changes in the tibialis anterior and soleus muscles. The treatment with vanillin had a proinflammatory effect in the tibialis anterior, which did not occur in the soleus, probably due to an increase in tissue blood flow and the ability of each muscle to respond to this alteration.
